# High-Frequency Resonance in the Gerbil Medial Superior Olive

**DOI:** 10.1371/journal.pcbi.1005166

**Published:** 2016-11-10

**Authors:** Jason Mikiel-Hunter, Vibhakar Kotak, John Rinzel

**Affiliations:** Center for Neural Science, New York University, New York, United States of America; Radboud Universiteit Nijmegen, NETHERLANDS

## Abstract

A high-frequency, subthreshold resonance in the guinea pig medial superior olive (MSO) was recently linked to the efficient extraction of spatial cues from the fine structure of acoustic stimuli. We report here that MSO neurons in gerbil also have resonant properties and, based on our whole-cell recordings and computational modeling, that a low-voltage-gated potassium current, I_KLT_, underlies the resonance. We show that resonance was lost following dynamic clamp replacement of I_KLT_ with a leak conductance and in the model when voltage-gating of I_KLT_ was suppressed. Resonance was characterized using small amplitude sinusoidal stimuli to generate impedance curves as typically done for linear systems analysis. Extending our study into the nonlinear, voltage-dependent regime, we increased stimulus amplitude and found, experimentally and in simulations, that the subthreshold resonant frequency (242Hz for weak stimuli) increased continuously to the resonant frequency for spiking (285Hz). The spike resonance of these phasic-firing (type III excitable) MSO neurons and of the model is of particular interest also because previous studies of resonance typically involved neurons/models (type II excitable, such as the standard Hodgkin-Huxley model) that can fire tonically for steady inputs. To probe more directly how these resonances relate to MSO neurons as slope-detectors, we presented periodic trains of brief, fast-rising excitatory post-synaptic potentials (EPSCs) to the model. While weak subthreshold EPSC trains were essentially low-pass filtered, resonance emerged as EPSC amplitude increased. Interestingly, for spike-evoking EPSC trains, the threshold amplitude at spike resonant frequency (317Hz) was lower than the single ESPC threshold. Our finding of a frequency-dependent threshold for repetitive brief EPSC stimuli and preferred frequency for spiking calls for further consideration of both subthreshold and suprathreshold resonance to fast and precise temporal processing in the MSO.

## Author Summary

Principal neurons of the medial superior olive (MSO) are fast and precise coincidence detectors involved in the neuronal computation of sound localization. We show that they exhibit resonance properties *in vitro*, responding to small oscillatory inputs, maximally at preferred frequencies. Their resonant frequencies are high, nearly a hundred-fold that found in cortical or hippocampal neurons. We used electrophysiological recordings (gerbil MSO) and computational modelling to identify the key biophysical factors for this fast resonance: a low-threshold potassium current, I_KLT_, whose activation time constant is slightly slower than the membrane time constant (*τ*_m_ < 1ms). An MSO neuron’s resonant frequency increases with stimulus amplitude and approaches the preferred frequency at which spiking is elicited with the lowest stimulus amplitude. This relationship between subthreshold and suprathreshold resonances also holds for trains of brief synaptic-like inputs in our neuron model, even though responses for weak subthreshold synaptic inputs are low-pass filtered. Our results demonstrate the important effects of stimulus amplitude and waveform, and I_KLT_, on measurements of impedance and therefore resonance. In addition, our data highlight the possibility that even brief synaptic input may be selectively filtered by the intrinsic biophysical properties of MSO neurons.

## Introduction

The intrinsic frequency preference of neurons for oscillatory inputs, otherwise known as subthreshold resonance, has been associated with the efficient extraction of auditory information. While turtle hair cells are highly tuned to pure tones up to 400Hz thanks to their intrinsic resonant properties [1], band-pass filtering of neurons in the frog midbrain and cricket auditory periphery improves AM frequency discrimination [2–3]. In mammals, principal neurons of the guinea pig medial superior olive (MSO) display an intrinsic preference for subthreshold, sinusoidal stimuli between 80-400Hz [4]. In simulations, this subthreshold resonance facilitates MSO neurons’ exquisite sensitivity to interaural time differences (ITDs) in the arrival time of low frequency sounds [4]: a key acoustic cue for the localization of low-frequency sounds along the horizontal axis [5–7]. In addition to its functional significance, the biophysical origins of this subthreshold resonance in the MSO neurons are of particular interest, especially given that resonant frequencies (sine-$f_{\text{res}}^{\text{sub}}$) are quite high, ~ 300 Hz, almost a hundredfold higher than the 4-12Hz range observed in hippocampal and neocortical neurons.

Subthreshold resonance emerges in a neuron from the interplay of its low- and high-pass filters to produce a peak in the impedance amplitude profile at a non-zero frequency [8–9]. While a neuron’s passive properties generate the low-pass filter with a corner frequency inversely proportional to its membrane time constant, the high-pass filter arises from one or more ‘resonant’, voltage-gated conductances providing negative feedback with a slower time course than that of the membrane. As coincidence detectors comparing tens of microseconds differences in the arrival times of a sound at either ear [5–7], MSO neurons require subthreshold millisecond membrane time constants to restrict the time window of integration for synaptic input [10–14]. While the fast passive properties are well characterized, there is less certainty about the ‘resonant’ current although Remme et al. [4] did highlight the low-threshold, voltage-gated potassium current, I_KLT_, as being an ideal candidate. This outward current not only provides negative feedback to depolarizing currents but also possesses suitable activation gating properties (time constant ≈ 1ms) and is present in high densities throughout an MSO neuron [14]. Additionally, experimental data from the mesencephalic nerve and computational evidence in the nucleus laminaris (the avian analogue of the mammalian MSO) point to I_KLT_ underlying similar high-frequency subthreshold resonances (>80Hz) [15–16].

Activation of I_KLT_ is also one of two negative feedback mechanisms, recruitable at subthreshold threshold voltage levels, that prevent MSO neurons and models from firing repetitively to steady or slowly-varying inputs; the other being the inactivation of the fast, sodium current, I_Na_ [17–18]. By opposing slow voltage changes (I_KLT_ activation) or limiting the availability of I_Na_ for spike initiation (inactivation of I_Na_), these mechanisms set a threshold that depends primarily on the rising slope rather than amplitude of the input, a distinguishing feature of phasic firing behavior or type III excitability [19–20]. Originally classified by Hodgkin [21], Type III excitability has more recently been described using reduced mathematical models [19–20]: opening up new possibilities to understand how phasic firing neurons are well suited for different encoding tasks. In the MSO, phasic firing has been studied extensively with respect to periodic stimuli as MSO neurons receive trains of inputs *in vivo* that are phase-locked to frequencies up to around 1kHz during ITD processing. When presented with sinusoidal or half-wave rectified stimuli of varying frequency, model MSO neurons display V-shaped, frequency-response maps with a preferred frequency (i.e. the input frequency at which the spike threshold is lowest) in the hundreds of Hertz [20,22]. In this study, we consider this to be a form of evoked spike resonance, where the resonant frequency ($f_{\text{res}}^{\text{spk}}$) is identified with the tip of the V-shaped response map. This band-pass filtering appears dependent on the voltage-gated activation of I_KLT_ as freezing the current’s conductance (so that it loses its voltage-gated properties) leads to low-pass properties as well as multiple spikes per cycles at low input frequencies (<80Hz) [20,22]. Outside of the MSO, I_KLT_ has also been implicated in determining spike timing of cortical pyramidal neurons which display their highest gain to noise input at 400Hz [23]. However the passive membrane properties of these cortical neurons are slow in comparison to the sub-millisecond time constant of MSO neurons [4, 10–14], therefore the encoding of fast components in noise input is restricted to suprathreshold responses [23].

Given the apparent importance of I_KLT_ to both subthreshold and suprathreshold regimes, could an MSO neuron’s subthreshold resonance actually influence its spike resonance and result in similar tuning, i.e. such that sine-$f_{\text{res}}^{\text{sub}}$ is close to sine-$f_{\text{res}}^{\text{spk}}$? Since studies characterizing subthreshold resonance have typically used small-amplitude sinusoidal currents to restrict the size of voltage responses and therefore limit the effect of membrane nonlinearities on impedance measurements [8,24–25], there is a gap in our understanding of how impedance profiles are affected by larger-amplitude sinusoidal stimuli or even stimuli with different waveforms like excitatory postsynaptic potentials (EPSCs). Indeed, considering the influence of the voltage-gated activation of I_KLT_ on suprathreshold responses [22], it is unlikely that their impedance profiles are invariant for all applied stimulus sizes or shapes. To address this issue, we studied subthreshold resonance in principal neurons of the gerbil MSO and neuron models using sinusoidal stimuli of different amplitude as well as trains of EPSCs (in the model). We have demonstrated, using dynamic clamp and both linear and nonlinear models, that I_KLT_ activation underlies a high-frequency subthreshold resonance in the gerbil MSO. Using an extended form of phase-plane analysis to analyze a reduced, two-variable model, we have shown that the amount and kinetics of I_KLT_ activation can also explain the significant increase in sine-$f_{\text{res}}^{\text{sub}}$ with stimulus amplitude that was observed experimentally. At perithreshold stimulus amplitudes i.e. close to spike threshold, the sine-$f_{\text{res}}^{\text{sub}}$ indeed was found to match the sine-$f_{\text{res}}^{\text{spk}}$, highlighting the close relationship between the two resonances in these phasic neurons. Trains of EPSCs with their fast, stereotyped rise times demonstrated the importance of stimulus waveform to measurements of impedance, promoting greater low-pass filtering of subthreshold synaptic stimuli (especially at weak EPSC amplitudes) compared to sinusoidal stimuli of equivalent amplitude. Nevertheless, a modest subthreshold resonance was generated by trains of stronger EPSCs and this translated into a spike resonance for EPSCs of sufficient amplitude, suggesting that brief, fast-rising EPSCs in a selective frequency range have a threshold for spikes lower than that of the single EPSC threshold in these slope-sensitive neurons.

## Methods

### Slice preparation

All experiments were approved by the New York University Animal Welfare Committee. Preweaned gerbils (*Meriones unguiculatus*), aged between 17 days postnatal (P17) and P24, were used to obtain transverse slices of 150–250 μm thickness. After an anesthetic (5% chloral hydrate) was administered and the animal was decapitated, its brainstem was dissected out and slices were cut in an oxygenated artificial CSF (ACSF) chilled to 4°C (in mM: 25 NaCl, 4 KCl, 1.2 KH_2_PO_4_, 1.3 MgSO_4_, 26 NaHCO_3_, 15 glucose, 2.4 CaCl_2_, and 0.4 L-ascorbic acid (pH 7.3 when bubbled with 95% O_2_-5% CO_2_)). The slices were then placed in an incubating bath containing oxygenated ACSF at 35°C for an hour before they were transferred to a holding dish maintained at room temperature.

### Whole-cell electrophysiology

Individual slices were selected from the holding dish and moved to the recording chamber after a minimum of 30 minutes post-incubation. A perfusion of oxygenated ACSF, heated to 32–37°C, flowed through the chamber at a constant rate of 1mL/min. Slices were held down by a Lycra/stainless steel harp to provide stability during recordings. The MSO principal neurons were visually identified with the Olympus BX50WI upright microscope using differential interference contrast and a 60x objective. Borosilicate patch pipettes (1.5mm outer diameter) were pulled with resistances between 3-5MOhm and backfilled with a K-gluconate, current clamp solution (in mM: 127.5 potassium gluconate, 0.6 EGTA, 10 HEPES, 2 MgCl_2_, 5 KCl, 2 ATP, 10 phosphocreatinine, and 0.3 GTP (pH 7.2)). Somatic recordings were low-pass filtered at 5kHz and acquired using an Axoclamp2A amplifier controlled by Labview 6.0 (sampling rate of 10kHz) through a NI PCI-6070E DAC card (National Instruments). Bridge compensation (for series resistance) and capacitance neutralization (for the pipette capacitance) were performed (>95%) to ensure a fast voltage transient (<0.1ms) [17]. No correction for liquid junction potential (≈-12mV) was performed. In whole-cell configuration, the cell’s viability was evaluated by checking that its resting potential was less than -40mV and action potentials could be elicited in response to suprathreshold depolarizing current injection. Its input resistance was measured by injecting a 100pA, hyperpolarizing current step. If at any time the series resistance rose above 20MOhm or the resonant properties of the neuron were found to have changed more than 10% from the previous recording in a similar condition, the data was discarded thereafter. 5mM 4-AP was added to the extracellular ACSF in a subset of experiments to probe the contribution of the low threshold potassium current, I_KLT_, to subthreshold resonance. 4-AP has previously been used in dynamic clamp experiments to block native I_KLT_ in the soma with minimal non-specific effects [26]. In these experiments, dynamic clamp was used to restore either a dynamic or frozen I_KLT_ to the MSO neurons. This was performed as previously described by Roberts et al. [26] but using gating parameters from Mathews et al. [14] and reversal potential set to -106mV (liquid-junction corrected to -94mV).

### Stimulus generation and analysis

**Fig 1:**
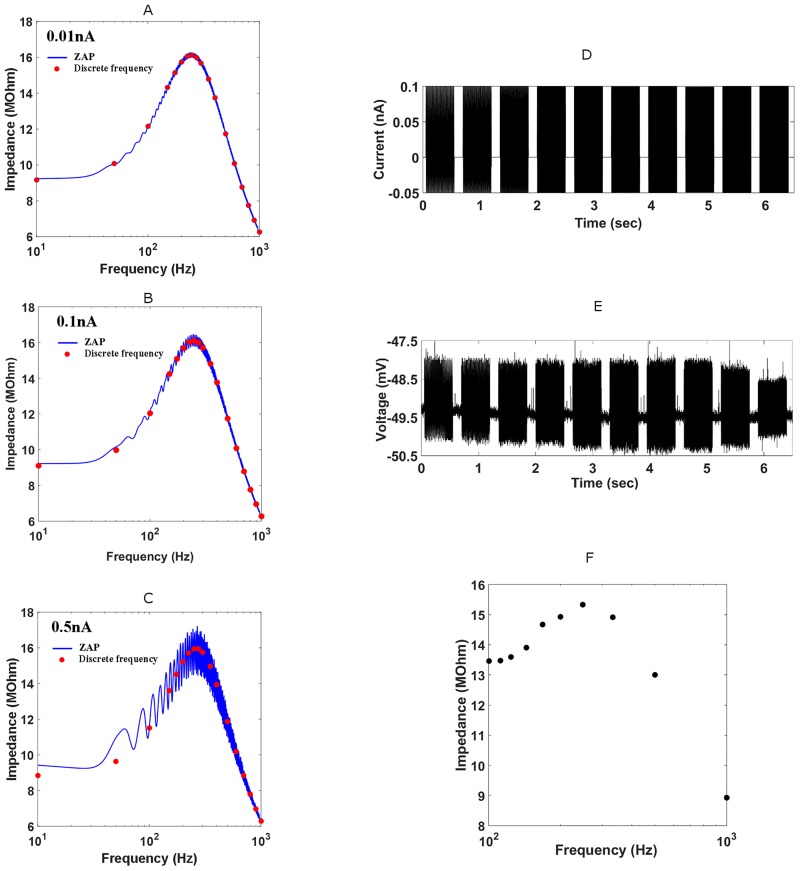
The discrete frequency protocol: its rationale and application. (A,B,C) Comparison of impedance profiles generated with ZAP and discrete frequency protocols at different stimulus amplitudes in the full nonlinear neuron model. For 0.01nA stimuli, impedance profiles generated using ZAP (blue) and discrete frequency (red dots) protocols overlapped. Both protocols covered a frequency range of 10-1000Hz; ZAP was linearly increasing over 1 second. At (B) 0.1nA and especially (C) 0.5nA stimulus strengths, the ZAP impedance profile diverged significantly from the discrete frequency one. These large oscillations in the ZAP impedance profile result from instantaneous impedance mesasurements being tested during hyperpolarizing and depolarizing phases of sinusoidal stimulus. (D, E, F) Example of discrete frequency protocol to measure impedance profile of MSO neuron experimentally. (D) Ten 0.1nA sinusoidal current inputs of increasing frequency (100Hz to 1000Hz) were injected somatically into an MSO neuron. The amplitude of the hyperpolarizing portion was halved. (F) Impedance profile was then generated using (E) voltage responses to sinusoidal input. DC current was injected to hold membrane potentials close to -48mV.

Most studies to date have utilized the ZAP current protocol (sinusoidal sweeps of linearly increasing frequency) to test subthreshold resonance in different neuronal types [4,8,24–25]. In the MSO, a 0.05nA ZAP stimulus (1 sec long) allows accurate measurements of impedance across frequencies from 10-1000Hz [4]; however, as the amplitude of the ZAP increases, larger inequalities between voltage deflections in the hyperpolarizing and depolarizing directions produce an impedance curve that oscillates with frequency (as shown by data from model in Fig 1A–1C). We therefore employed sinusoids of single, discrete frequencies whose hyperpolarizing portion was multiplied by a factor of 0.5 to give accurate impedance measurements for stimuli of all amplitudes. A 0.5 factor was applied to study a larger range of subthreshold stimuli strength as hyperpolarizing an MSO neuron has been shown to increase the probability of a subsequent depolarizing current generating a spike [27]. This ‘discrete frequency’ protocol consisted of ten 500ms sinusoids of ascending frequency with 250ms pauses between each sinusoid (Fig 1D). The frequencies were initially distributed widely to discover whether a neuron was indeed resonant before they were focused around the peak of this resonance. The minimum difference between frequencies was between 4-10Hz. After generating the stimuli and then recording the voltage response in Labview 6.0 (Fig 1E), analysis and plotting of impedance curves were performed in MATLAB (Fig 1F). The sine-$f_{\text{res}}^{\text{sub}}$ was considered the input frequency at which the peak impedance value was located. If this was not clear however and a broad peak was observed, then the mean input frequency of this broad peak was taken as the sine-$f_{\text{res}}^{\text{sub}}$. Three groups of subthreshold stimulus strengths were used: low subthreshold (0.05–0.15nA) stimulus, a mid-subthreshold (0.8nA) stimulus and a perithreshold stimulus (0.95nA-1.2nA). A perithreshold stimulus was considered to be the largest stimulus amplitude that evoked subthreshold responses and was calculated for each neuron individually (typically 50-150pA below spike threshold).

When characterizing the sine-$f_{\text{res}}^{\text{sub}}$ or using dynamic clamp to demonstrate the contribution of I_KLT_ to this subthreshold resonance (both experiments using 0.05–0.15nA stimuli), analysis was performed using Fast Fourier transforms (FFT) to attain the power spectra of both the current stimulus and voltage responses [4,8,24–25]. Impedances were then calculated by dividing the voltage FFT by the current FFT and taking the square root at each frequency. The nonlinearity of voltage responses to large amplitude stimuli biases the calculation of sine-$f_{\text{res}}^{\text{sub}}$ by power spectrum analysis towards higher frequencies. We therefore used maximum difference between local voltage peaks and the adjacent minima, or Max-min, divided by the peak-to-peak sinusoidal current as a measurement at each stimulus frequency instead for large amplitude stimuli [28]. This was used exclusively when determining the effect of different stimulus amplitudes on subthreshold resonant properties. Sine-$f_{\text{res}}^{\text{sub}}$ (the sinusoidal input frequency at which the maximum peak impedance was observed in an impedance profile) and the Q factor (a measure of how resonant a neuron was, calculated by dividing the max impedance by the input resistance) were calculated from the impedance profiles. Analysis of suprathreshold responses (both for the electrophysiological and model data) consisted of taking the differential of the voltage response and using an absolute threshold for action potentials of -110 mV/ms (representing the steep downward slope of the action potential following its peak as a result of strong hyperpolarization by both I_KLT_ and I_KHT_). The number of action potentials was then divided by the number of cycles during each 500ms presentation to give the spike probability per cycle. The sine-$f_{\text{res}}^{\text{spk}}$ was considered the sinusoidal input frequency at which the lowest input amplitude could generate spiking. When analyzing grouped data, the mean and standard error of the mean were displayed. A significance criterion of p<0.05 was used when performing statistical tests on the grouped data.

### Model simulations

We used a single compartment model developed for the MSO [14,29]. The gating kinetics for the subthreshold conductances, I_KLT_ and I_H_ (hyperpolarization-activated mixed-cation current) were derived from Mathews et al. [14] and Khurana et al. [29] respectively. A spiking mechanism was added from a model of bushy cell in the anterior ventral cochlear nucleus (AVCN) [30]. The current balance equation therefore included I_KLT_, I_H_, I_KHT_ (high-threshold potassium current), I_Na_ (fast sodium current) as well as I_lk_, (voltage-independent, leak current).

$$C_{m}\frac{dV}{dt}=-I_{\text{Na}}-I_{\text{KHT}}\;-I_{\text{H}}-I_{\text{KLT}}-I_{\text{lk}}+I\left(t\right)=\;-{\bar{g}}_{\text{Na }}m^{3}h\left(V-E_{\text{Na}}\right)-{\bar{g}}_{\text{KHT}}\left(0.85n^{2}+0.15p\right)\left(V-E_{\text{K}}\right)-{\bar{g}}_{\text{H}}\left(k_{r}r_{f}+\left(1-k_{r}\right)r_{s}\right)\left(V-E_{\text{H}}\right)-{\bar{g}}_{\text{KLT}}w^{4}z\left(V-E_{\text{K}}\right)-{\bar{g}}_{\text{lk}}\left(V-E_{\text{lk}}\right)+\;I\left(t\right)$$ (1)

Where V is the membrane potential and C_m_ is the capacitance (25pF) and where *I*(*t*) is calculated as follows:

$$I\left(t\right)=A\beta \text{sin}\left(2\pi f_{m}t\right)$$ (2)

Where *f*_*m*_ is the stimulus frequency, *A* is the peak stimulus amplitude in nA and β = 0.5 if sin(2*πf*_*m*_*t*) < 0 otherwise β = 1. The maximum channel conductances were: *ḡ*_Na_ = 1,275 nS, *ḡ*_KHT_ = 150 nS, *ḡ*_KLT_ = 190nS, *ḡ*_H_ = 70nS, and *ḡ*_lk_ = 15 nS; reversal potentials were *E*_Na_ = +55 mV, *E*_K_ = –106 mV, *E*_H_ = –37 mV, and *E*_lk_ = –77.5 mV the factor k_r_ = 0.65. The value of *ḡ*_Na_ was reached by roughly matching the sinusoidal stimulus amplitude threshold required to attain suprathreshold results *in vitro*. The resting membrane potential of the model, *V*_rest,_ was -58mV. The input resistance of the model was 8.5MOhm at rest. The membrane time constant (*τ*_m_) was 0.34ms. The dynamics for the gating variables were described by the following differential equation:

$$\frac{dx}{dt}=\frac{x_{\infty }\left(V\right)-x}{\tau_{x}\left(V\right)}$$ (3)

where *τ*_*x*_(*V*) is the time constant of the variable *x = w*, *z*, *r*_*f*_, *r*_*s*_, *n*, *p*, *m* and *h* and *x*_∞_(*V*) is the steady state value of *x* and voltage, *V*. The expressions for *τ*_*x*_(*V*) and *x*_∞_(*V*) were either taken from the voltage-clamp studies of Khurana et al. [29] or Rothman and Manis [30]. As the time constants for I_KLT_ (*τ*_*w*_, *τ*_*z*_) and I_H_ (*τ*_*rf*_, *τ*_*rs*_) were natively fit at 35°C by Khurana et al. [29], it was necessary to shorten the remaining time constants (for *I*_Na_ and *I*_KHT_) by a factor of 0.24 (Q10 = 3 [29]) to emulate this temperature in the model. For a subset of simulations, we froze one or more of the conductances gating variable, *x* to their resting values, *x*_∞_(*V*_rest_), where *V*_rest_ = -58mV.

In addition to the single compartment model with full gating kinetics, we also used a linearized version of our model with free gating parameters reduced to activation (*w* gating) of I_KLT_ with all other gating properties frozen at *V*_rest_. The linearization was performed as described by Khurana et al. [29] to give an explicit equation that predicted impedance *Z* at time *t* and voltage *V* in response to small amplitude stimuli:

$$Z\left(t,V;\omega \right)\;=\;{\left[i\omega C_{\text{m}}+g_{\text{m}}+\frac{g_{\text{w}}\left(t,V\right)}{\left(1+i\omega \tau_{\text{w}}\left(V\right)\right)}\right]}^{-1}$$ (4)

where $g_{\text{m}}=\;{\bar{g}}_{\text{KLT}}w_{\infty }{}^{4}z_{\infty }+\;{\bar{g}}_{\text{lk}}+\;{\bar{g}}_{\text{KHT}}\left(0.85n_{\text{fro}}{}^{2}+0.15p_{\text{fro}}\right)\;+\;{\bar{g}}_{\text{H}}\left(k_{r}r_{f}{}_{\text{fro}}+\left(1-k_{r}\right)r_{s}{}_{\text{fro}}\right)\;+\;{\bar{g}}_{\text{Na }}m_{\text{fro}}{}^{3}h_{\text{fro}}$ and $g_{\text{w}}=4\;{\bar{g}}_{\text{KLT}}w_{\infty }{}^{3}\left(V\right)z\left(t\right)\left(V-E_{\text{K}}\right){w^{\prime }}_{\infty }\left(V\right)$, where *ω* = 2*πf* for frequency *f* and *w’*_*∞*_(*V*) is the derivation of the activation function *w*_*∞*_(*V*) with respect to V evaluated at V and *x*_fro_ represents conductance gating variable, *x*, frozen to its resting value, *x*_∞_(*V*_rest_), where *V*_rest_ = -58mV

An adapted version of the ‘discrete frequency’ protocol was employed to stimulate the model neuron. A single frequency was tested at a time using a 1 second long control sine-wave although the sinusoid was still multiplied by factor of 0.5 in the hyperpolarizing direction. Given that Khurana et al. [29] encountered adaptation of the two main subthreshold currents, I_KLT_ and I_H_, over the course of hundreds of milliseconds, at least 1.5 seconds of quiescence was introduced before the sinusoidal stimulus started and impedance measurements were calculated using the last 500ms of the stimulus to get the steady-state response. Analysis was performed in a similar manner to the recordings from brain slices using power spectrum analysis and Max-min measurements computed in MATLAB.

In addition to finding the $f_{\text{res}}^{\text{sub}}$ for sinusoidal current input, we also performed the same task in the model using excitatory synaptic input. One second trains of simulated excitatory synaptic inputs were implemented at different frequencies; each individual synaptic event was modelled as an α function current input:

$$I_{syn}\left(\text{t}\right)\;=\text{ A }\frac{t-\;t_{0}}{\tau_{syn}}\text{exp}\left(1-\frac{t-\;t_{0}}{\tau_{syn}}\right)$$ (5)

Where A is the peak amplitude; *τ*_*syn*_ is the time constant describing the rise and decay of the synaptic input and *t*_0_ is the onset of the synaptic event. *τ*_*syn*_ was set at 0.3ms based on *in vitro* experimental data [14,31–32]. EPSC-$f_{\text{res}}^{\text{sub}}$ was considered the input train frequency at which the largest Max-min difference was observed for the last 5 EPSCs of a particular subthreshold amplitude. EPSC-$f_{\text{res}}^{\text{spk}}$ was the input train frequency at which spike threshold was observed; spike thresholding for EPSCs was performed in the same manner as for sinusoidal stimuli.

### Phase plane analysis

Phase plane analysis is a graphically-based tool used to visualize the geometrical properties of dynamical systems and their solutions. We applied phase plane analysis to the voltage responses of a reduced model with only two free variables, *w* and *V* (all other gating variables except those for I_Na_ and I_KHT_ were frozen at their *V*_rest_ values. I_Na_ and I_KHT_ were removed altogether as their contribution was negligible when frozen), to study how activation of I_KLT_ influenced the voltage responses at different sinusoidal stimulus amplitudes. This has previously been implemented successfully by Rotstein [28] to explain subthreshold resonance in linear and quadratic models. We plotted *V*-*w*^*4*^ trajectories that represent the final cycle of a response to a sinusoidal stimulus at a particular frequency and stimulus amplitude. The flow along a trajectory was counterclockwise. The fourth power of *w* was plotted as it determined the instantaneous conductance (fraction of maximum value) for I_KLT_ and therefore better represented its activation. The *V*-nullcline and *w*-nullcline were the curves along which $\frac{dV}{dt}\;=\;0$ and $\frac{dw}{dt}\;=\;0$. For the *V*-nullcline we solved for *w*^4^ as a function of V in Eq (1) with $\frac{dV}{dt}\;=\;0$. From the gating dynamics for *w* we obtained the *w*-nullcline, expressed in terms of *w*^4^ versus *V*: *w*^4^ = [*w*_*∞*_(*V*)]^4^. All parameters took the values as stated above in Eq (1).

## Results

### Subthreshold resonance in the gerbil

**Fig 2:**
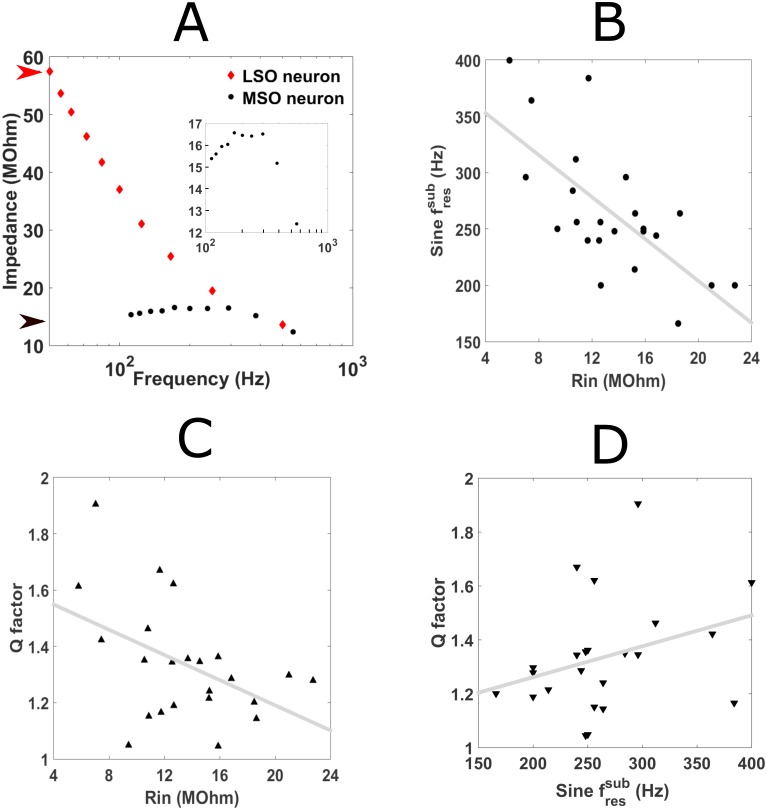
Subthreshold resonance in gerbil MSO and its origins. (A) Plot of impedance profiles of resonant medial superior olive (MSO) neuron (black dots) and non-resonant lateral superior olive (LSO) principal neuron (red dots). Arrows on impedance axis represent input resistance of MSO (black arrow, 14.25MOhm) and LSO (red arrow, 59.3MOhm). (A inset) The sine-$f_{\text{res}}^{\text{sub}}$ (212Hz) is shown for the MSO neuron from (A). (B) Graph of sine-$f_{\text{res}}^{\text{sub}}$ versus R_in_ for all gerbil MSO neurons. Linear regression gave a line of best fit with the following equation: *y* = −9.31*x* + 390.2. (C) Q-factor versus sine-$f_{\text{res}}^{\text{sub}}$ for all gerbil MSO neurons. Linear regression gave a line of best fit with the following equation: *y* = −0.02*x* + 1.638. (D) Q factor versus R_in_ for all gerbil MSO neurons. Linear regression gave a line of best fit with the following equation: *y* = 0.001*x* + 1.04.

To characterize the subthreshold filters in P17-24 gerbil MSO principal neurons, whole-cell recordings in 23 animals at near physiological temperatures were obtained (average age, P19.5 ± 0.4, n = 23) (see Methods). Resting membrane potential (mean mV, -45.4 ± 1.1; values were not corrected for liquid junction potential) and input resistance (R_in_) (mean MΩ, 13.97 ± 0.91, n = 23) were measured while a phasic response to suprathreshold stimuli was also ensured. Small amplitude (0.05–0.15nA) sinusoidal currents were injected at discrete frequencies to probe subthreshold resonance (Fig 1D). The impedance profile across frequencies (Fig 1F) was then computed by dividing the power spectrum of the resulting voltage response by that of the stimulus (Fig 1E). A neuron was considered resonant if its peak impedance was larger than its input resistance. Quantification of the resonant peak was performed by measuring the sine-$f_{\text{res}}^{\text{sub}}$ i.e. frequency associated with peak impedance, and Q factor, i.e. the degree of the resonance calculated as the peak impedance divided by the input resistance. Fig 2A shows an example impedance profile for a ‘band-pass’ cell compared to an adjacent lateral superior olive (LSO) neuron which displayed no resonant peak and was therefore considered ‘low-pass’.

All MSO neurons displayed subthreshold resonance with an average sine-$f_{\text{res}}^{\text{sub}}$ of 266.35±11.9Hz, (n = 23) and an average Q factor of 1.34±0.04 (n = 23). This demonstrated that the subthreshold resonance was high frequency but only slightly underdamped i.e. not especially frequency selective. There was no indication that either property was associated with position of the MSO neuron along the tonotopic axis or the age of the animal. Both sine-$f_{\text{res}}^{\text{sub}}$ and Q factor displayed a significant negative correlation with input resistance (Fig 2B and 2C) (R_in_ vs. sine-$f_{\text{res}}^{\text{sub}}$: R^2^ = 0 .475, F(1, 21) = 19, p = 0.00028; R_in_ vs. Q factor: R^2^ = 0 .214, F(1, 21) = 5.72, p = 0.026). This suggested that the intrinsic conductances contributing to the low input resistance also helped shape its impedance profile and therefore defined its subthreshold resonance. By contrast, we only found a weak correlation between the sine-$f_{\text{res}}^{\text{sub}}$ and Q factor (Fig 2D) (R^2^ = 0 .103, F(1, 21) = 2.41, p = 0.136, ns).

### I_KLT_ plays an important role in subthreshold resonance

**Fig 3:**
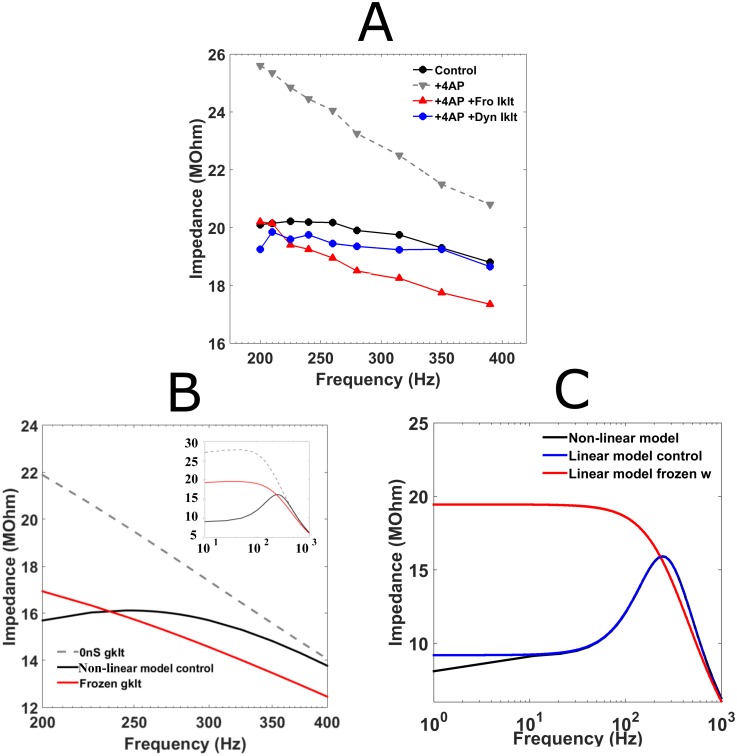
I_KLT_ underlies the subthreshold resonance in the gerbil MSO. (A) Impedance profile of resonant MSO neuron (black circles) displayed high impedance and low-pass properties after 4-AP was applied (red inverted triangles). Adding a simulated g_KLT_ (dynamic clamp) with voltage-gated parameters (red circles) returned resonance to the neuron; however if the simulated conductance’s gating properties were frozen at resting potential (red righted triangles), the impedance profile shifted down to normal values but low-pass properties remained. (B and inset) Impedance profiles generated with a nonlinear model neuron also demonstrated that I_KLT_ was necessary for resonance (solid black line). Removing g_KLT_ altogether (dashed black line) or freezing g_KLT_ (solid red line) led to low-pass properties. Note that freezing g_KLT_ also shifted the impedance profile to lower values. (C) Impedance profile for the nonlinear model (solid red line) matched those of a linearized model where the only free parameter was activation of g_KLT_ (dotted black line). Freezing g_KLT_ activation gating parameter produced non-resonance (dotted blue line).

In order to determine the role of the low-threshold voltage-gated potassium current, I_KLT_, in this resonance, a broad-spectrum potassium channel antagonist 4-AP was used. Fig 3A shows an example MSO neuron trace in which the neural filter changed from band-pass (black circles, Fig 3A) to low-pass (red inverted triangles, Fig 3A) and impedances increased for all frequencies tested after 4-AP application (this was true for all 3 MSO neurons tested). The subthreshold resonance was restored when dynamic-clamp was employed to introduce a simulated (or hybrid) I_KLT_ to the MSO neurons (red circles, Fig 3A) (see Methods: simulated I_KLT_ conductance, g_KLT_, according to an MSO-based current described by Mathews et al. [14] [Roberts et al. [26]). The amount of g_KLT_ applied in each MSO neuron was found by matching both resting potential and input resistance before 4-AP had been administered (mean g_KLT_ added, 215±12.6nS, n = 3). To highlight the importance of the voltage-gated properties of I_KLT_ to the subthreshold resonance, we again used dynamic clamp to now introduce a frozen g_KLT_ (red righted triangles, Fig 3A). Similar conductance levels were applied for the frozen g_KLT_ as for the fully dynamic conductance. Although the addition of frozen g_KLT_ led to a decrease in impedances across all frequencies, subthreshold resonance was not restored and the three MSO neurons tested remained low-pass.

To further confirm that g_KLT_ was involved in this subthreshold resonance, we performed simulations with a neuron model based on the Rothman and Manis [30] model of guinea pig cochlear nucleus bushy cells but with revised current densities and kinetic properties of the two principal conductances at rest to match properties of gerbil MSO neurons [14,29]: I_KLT_, and the hyperpolarization activated cation conductance, I_H_. The results of the simulations matched the experimental observations: while the model neuron demonstrated subthreshold resonance under control conditions, using a frozen g_KLT_ (red trace, Fig 3B) or removing the conductance altogether (dashed grey trace, Fig 3B) caused the neuron model to act as a low-pass filter. In addition, freezing g_KLT_ also led to impedance values that more closely matched the resonant model neuron than the model with I_KLT_ absent (Fig 3B). To confirm that it was the activation of I_KLT_ which provided the necessary negative feedback for subthreshold resonance to occur, we implemented a reduced, linear model in which all gating variables apart from I_KLT_ activation, *w*, were frozen (dotted black trace, Fig 3C). The impedance curve, generated analytically for this linear model, was found to match that of the model with full kinetics, demonstrating that the activation of I_KLT_ (and its time course) was vital to the high frequency subthreshold resonance encountered in these gerbil MSO principal neurons.

### Sine-$f_{res}^{sub}$ increases with stimulus amplitude

**Fig 4:**
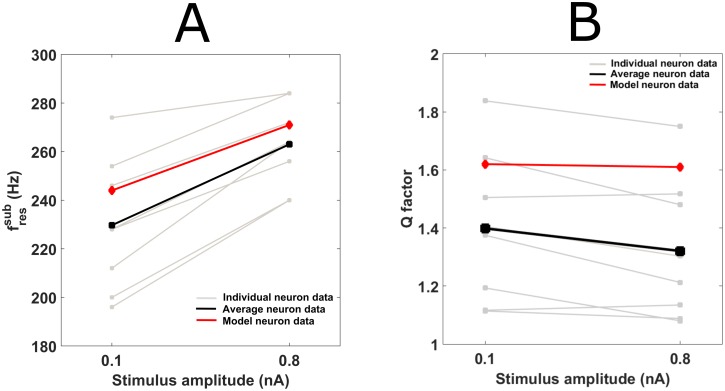
Effects of increasing stimulus amplitude on resonant properties. (A) Increasing stimulus amplitude from 0.1nA to 0.8nA caused sine-$f_{\text{res}}^{\text{sub}}$ to increase both experimentally (individual neurons in grey, average in black) and in model (red). (B) At the same time, the Q factor dropped both experimentally (individual neurons in grey, average in black) and in model neuron (red).

While small amplitude stimuli are typically chosen for characterizing subthreshold resonance at rest, the resulting band-pass filter may not prove invariant to changes in stimulus amplitude. Stimulus amplitudes of 0.1nA (to measure subthreshold resonance at rest) and 0.8nA (to measure it for more depolarizing stimuli) were therefore presented to the MSO neurons to compare sine-$f_{\text{res}}^{\text{sub}}$ and Q factor. Sine-$f_{\text{res}}^{\text{sub}}$ increased significantly *in vitro* from 227.6±6.8 Hz (n = 8) for 0.1nA stimuli to 257.5±3.6 Hz (n = 8) for 0.8nA stimuli (Paired t-test p<0.01, n = 8) (Fig 4A). The accompanying decrease in Q factors equally proved significant (mean for 0.1nA = 1.40±0.09, mean for 0.8nA = 1.32±0.09; Paired t-test p = 0.00016, n = 8) (Fig 4B). The two stimulus strengths were also tested in the full neuron model. The sine-$f_{\text{res}}^{\text{sub}}$ increased from 244Hz at 0.1nA to 271Hz at 0.8nA (Fig 4A): an 11.07% percentage change that is very similar to the 15.13±2.5% observed for the paired data (n = 8). The Q factor, on the other hand, decreased from 1.62 to 1.61 (Fig 4B); a 0.6% percentage decrease that is almost unchanged compared to the 5.42±1.78% decrease observed *in vitro* for paired data (n = 8).

### I_KLT_ activation can explain shifts in sine-$f_{res}^{sub}$ caused by varying stimulus parameters

**Fig 5:**
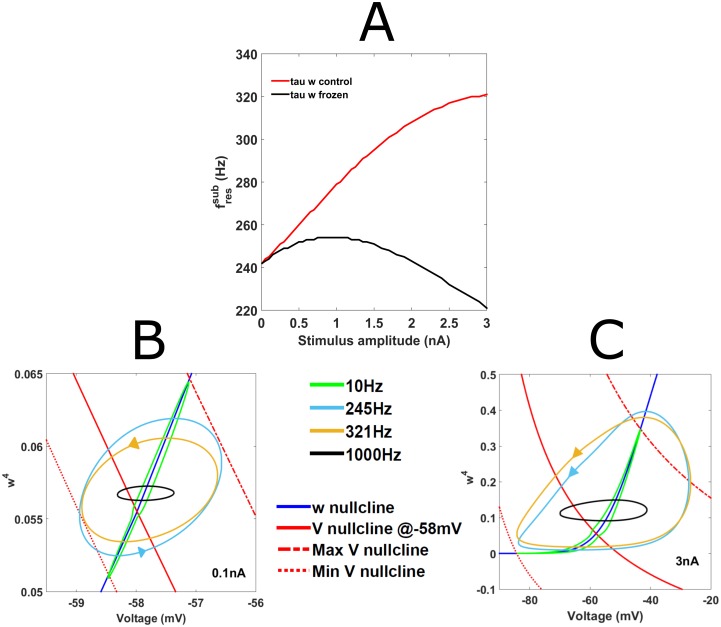
Increase in sine-$f_{\text{res}}^{\text{sub}}$ with stimulus amplitude can be explained by differences in I_KLT_ activation. (A) Graph of sine-$f_{\text{res}}^{\text{sub}}$ versus stimulus amplitude for a reduced nonlinear model where only *w-*gating is free (Control voltage-gated *τ*_w_ (red); Frozen *τ*_w_ (black)). Sine-$f_{\text{res}}^{\text{sub}}$ increased to a maximum of 321Hz at 3nA when *τ*_w_ was voltage-gated but plateaued at 254Hz when *τ*_w_ was frozen at a *V*_*rest*_. (B, C) Phase plane representation of dynamical responses (see Methods). *V-w*^*4*^ phase plane trajectories for 0.1nA (B) and (C) 3nA sinusoidal inputs presented to the reduced nonlinear model above. Stimulus frequencies shown are: 10Hz (green); 245Hz (turquoise); 321Hz (golden) and 1000Hz (black). *V*-nullcline at rest (solid red) i.e. when *I(t)* = 0nA; *V*-nullcline at minimum sinusoidal currents (large dashed red) i.e. when $I\left(t\right)\;=\;\frac{-A}{2}$; *V* nullcline at maximum sinusoidal current (mixed dash red) i.e. when *I*(*t*) = *A* and *w*-nullcline (blue).

An adapted form of phase-plane analysis, developed by Rotstein and Nadim [33] to examine subthreshold resonance in a two-dimensional system, was implemented here to determine whether I_KLT_ activation could explain the increase in sine-$f_{\text{res}}^{\text{sub}}$ with stimulus amplitude. For consideration of subthreshold resonance we ignored and removed I_Na_ and I_KHT_ from the model and froze all other gating variables except activation of I_KLT_, *w*. This reduced version therefore had only two dynamic variables, *V* and *w*, and we visualized its behavior by plotting responses in the *V-w*^*4*^ plane (Fig 5B and 5C). This allowed us to observe how the variations in sinusoidal current stimulus and I_KLT_ activation influenced the voltage trajectory across a stimulus cycle.

In order to make the geometric comparison of voltage trajectories at different frequencies clearer, we used two stimulus amplitudes that were well-separated (0.1nA and 3nA) and hence produced sine-$f_{\text{res}}^{\text{sub}}$ that reflected this difference (245Hz and 321Hz respectively for sinusoidal stimuli of 0.1nA and 3nA amplitudes (Fig 5A)). In addition to these two sine-$f_{\text{res}}^{\text{sub}}$, we included 10Hz and 1000Hz as they represented extreme frequencies in the impedance curve and could help us to understand what was happening away from the resonant peak.

Increasing the stimulus amplitude from 0.1nA (Fig 5B) to 3nA (Fig 5C) had little effect on the shape of the *V-w*^*4*^ trajectories for the 10Hz (green traces) and 1000Hz (black traces) inputs although it was naturally conducive to larger voltage maxima and minima being observed. At 10Hz, the voltage trajectories tracked the *w*-nullcline closely in both Fig 5B and 5C, suggesting that I_KLT_ activation was able to follow this slow input and curtail the changes in voltage it caused during its depolarizing phase. At 1000Hz, the voltage trajectories in both Fig 5B and 5C were constrained to a small-amplitude, horizontal profile as they trailed the rapidly moving V-nullcline which proved too fast for I_KLT_ activation. For extremely high frequencies (>10kHz), we would expect the trajectory to shrink down further towards a point on the intersection of the *V-* and *w*- nullclines. At intermediate frequencies however, the difference between the two stimulus amplitudes was apparent. At 0.1nA (Fig 5C), the *V-w*^*4*^ trajectory at the resonant frequency 245 Hz (turquoise trace) best bridged the two trajectory profiles observed for 10Hz and 1000Hz, producing the largest voltage difference between peak and minimum voltage and therefore the peak impedance. By comparison, the *V-w*^*4*^ trajectory for 321Hz (golden trace) at 0.1nA possessed a more compact profile beginning to resemble the 1000Hz trajectory profile more closely. At 3nA (Fig 5C), *V-w*^*4*^ trajectories covered a much larger range of values on both axes and were clearly more nonlinear than their 0.1nA counterparts. However the 245Hz *V-w*^*4*^ trajectory profile (turquoise trace) now followed the *w*-nullcline more closely and therefore extended less far along the voltage axis compared to the 321Hz stimulus (golden trace). As *τ*_w_ decreases by a factor of 3–5 at higher voltages (*τ*_w_ at *V*_rest_ = 1.51ms; *τ*_w_ at -30mV = 0.44ms), the increased depolarization caused by this stronger stimulus led to faster and larger I_KLT_ activation as demonstrated by the *V-w*^*4*^ trajectory reaching higher values of *w*^*4*^ while also tracking the *w*-nullcine more closely (Fig 5C). Consequently the model for 3nA could therefore track the higher frequency input better, ensuring the larger voltage deviation and peak impedance at this increased sine-$f_{\text{res}}^{\text{sub}}$, 321 Hz. To confirm that the voltage-dependent *τ*_w_ contributed to the increase in sine-$f_{\text{res}}^{\text{sub}}$, we froze the variable at *V*_rest_ (*τ*_w_ = 1.51ms) and plotted sine-$f_{\text{res}}^{\text{sub}}$ at different stimulus amplitudes (Fig 5A). Sine-$f_{\text{res}}^{\text{sub}}$ increased more slowly with stimulus amplitude after *τ*_w_ was frozen, plateauing at 254Hz (0.75nA) before decreasing at larger amplitudes.

### Sine-$f_{res}^{sub}$ closely matches the sine-$f_{res}^{spk}$ in gerbil medial superior olive

**Fig 6:**
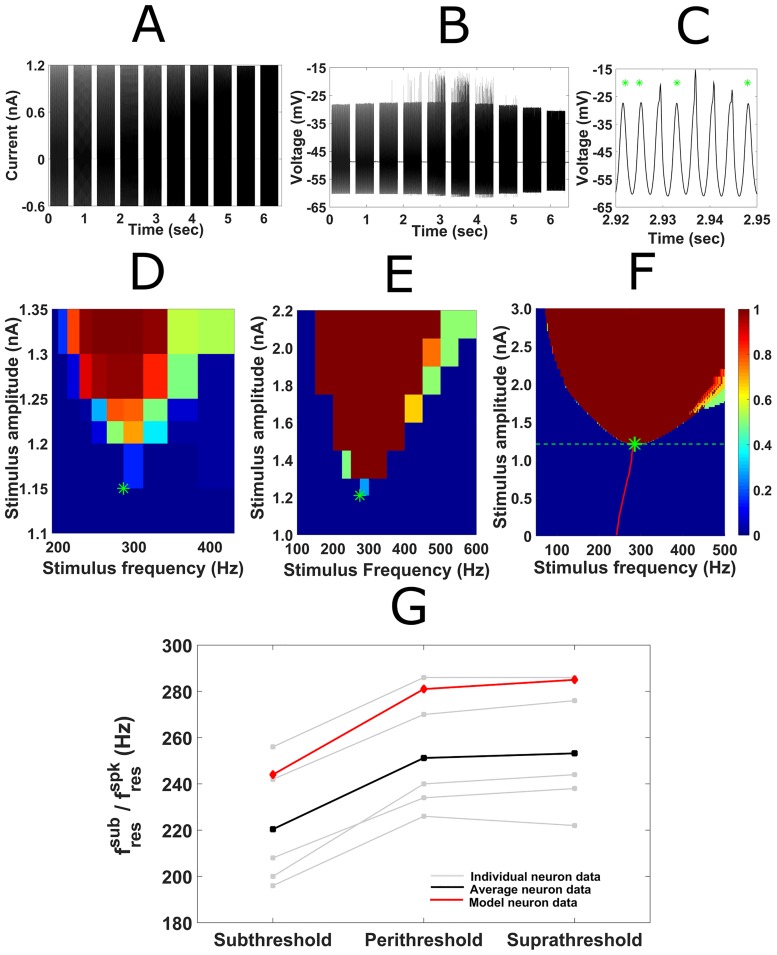
Comparing subthreshold and spike resonant peaks, and regions of spike entrainment. (A) Example of discrete frequency protocol used to measure suprathreshold responses experimentally. Ten 1.2nA sinusoidal current inputs of increasing frequency (250Hz to 500Hz) were injected somatically into an MSO neuron (amplitude halved in hyperpolarizing direction). (B) Corresponding voltage response of MSO neuron to sinusoidal stimuli in (A). (C) Expanded view of voltage response in (B) to 333Hz sinusoidal stimulus demonstrates the difference between spikes and subthreshold responses (green asterisks). (D) A suprathreshold response map for an example MSO neuron displayed a sine-$f_{\text{res}}^{\text{spk}}$ at 275Hz (green asterisk). The color represents the spike probability per cycle. (E) An equivalently sampled heat map for full, nonlinear model shows a similar asymmetric V-shape and sine-$f_{\text{res}}^{\text{spk}}$ of 280Hz (green asterisk). (F) Response map combining subthreshold (below 1.19nA) and suprathreshold responses (above 1.19nA) to sinusoidal stimuli in full, nonlinear neuron model. Spike threshold is displayed by the horizontal dashed green line. Subthreshold resonance is shown by the red curve; the sine-$f_{\text{res}}^{\text{spk}}$ is shown by the green asterisk. Note that the largest sine-$f_{\text{res}}^{\text{sub}}$ (281Hz) closely matched the sine-$f_{\text{res}}^{\text{spk}}$ (285Hz, green asterisk). (G) The perithreshold sine-$f_{\text{res}}^{\text{sub}}$ and sine-$f_{\text{res}}^{\text{spk}}$ closely matched in both the grouped MSO data (mean in black; individual neurons in grey) and the simulation data (red).

In order to measure the sine-$f_{\text{res}}^{\text{spk}}$ of individual gerbil MSO neurons, we used the discrete frequency protocol to measure spike probability per cycle across a range of sinusoidal stimulus frequencies and amplitudes (Fig 6A, 6B and 6C). Heat-maps for the five MSO neurons tested were all strongly V-shaped, with a mean sine-$f_{\text{res}}^{\text{spk}}$ of 252.80±12.1Hz (n = 5) and a mean threshold amplitude of 1.15±0.02nA (n = 5) (see example heat map in Fig 6D). We compared this to the subthreshold resonance of these MSO neurons at two amplitudes: low subthreshold (0.05–0.15nA) and perithreshold (0.95–1.2nA). The sine-$f_{\text{res}}^{\text{spk}}$ differed markedly from the mean sine-$f_{\text{res}}^{\text{sub}}$ frequency at low subthreshold amplitudes (220.80±10.8Hz, Paired t-test p = 0.00043, n = 5), but was not significantly different from the mean sine-$f_{\text{res}}^{\text{sub}}$ for perithreshold stimuli (260±13.7Hz; Paired t-test p = 0.33, n = 5) (Fig 6G).

Simulations from the full dynamic model agreed well with these electrophysiological data. The heat-map for the model was also asymmetrically V-shaped with a sine-$f_{\text{res}}^{\text{spk}}$ of 285Hz and a stimulus threshold of 1.195nA (Fig 6E and 6F). Sine-$f_{\text{res}}^{\text{sub}}$ also increased monotonically with stimulus amplitude at low to medium subthreshold values; before plateauing at a value of 281Hz at the largest subthreshold stimulus amplitude.

### Resonant properties differ for repetitive brief synaptic-like inputs

**Fig 7:**
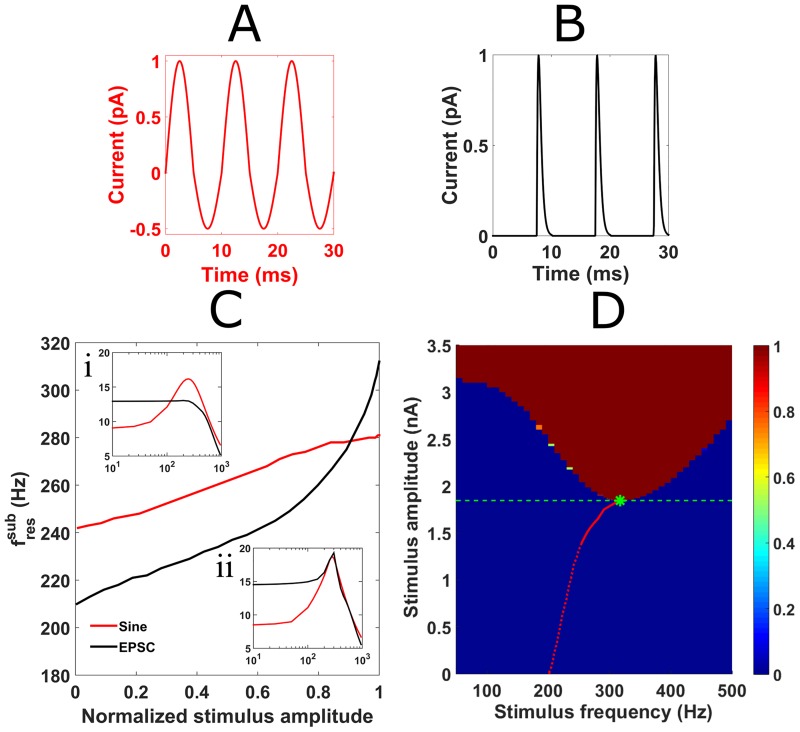
Effect of synaptic input on subthreshold and spike resonance in the neuron model. (A) An example 100Hz sinusoidal stimulus. (B) An example 100Hz train of simulated EPSCs. (C) Comparing subthreshold resonance for sinusoidal and synaptic trains of inputs required normalization of stimulus amplitude as the input threshold for spiking increased from 1.19nA for the sinusoidal input to 1.84nA for the synaptic input. Using excitatory synaptic current inputs (black) led to a larger range of $f_{\text{res}}^{\text{sub}}$ than using sinusoidal stimuli (red) (202Hz to 316Hz for EPSCs compared to 242Hz to 281Hz for sinusoidal stimuli). (Inset i and ii) Impedance profiles for sinusoidal (red) and synaptic (black) stimulus at 0.1 (inset i) and 0.95 (inset ii) normalized stimulus strength demonstrate that the Q factor was consistently larger for sinusoidal stimuli (red curve) compared to EPSCs (black curve) especially at low stimulus amplitudes. (D) Response map combining subthreshold (below 1.84nA) and suprathreshold responses (above 1.84nA) to trains of EPSCs in neuron model. Spike threshold is displayed by the horizontal dashed green line. EPSC-$f_{\text{res}}^{\text{sub}}$ is shown by the red curve; the EPSC-$f_{\text{res}}^{\text{spk}}$ is shown by the green asterisk. Where Q factor was smaller than 1.1 for the subthreshold resonance (<1.4nA), the red line is dashed and the impedance profile is effectively low-pass. The largest EPSC-$f_{\text{res}}^{\text{sub}}$ (316Hz) closely matched the EPSC-$f_{\text{res}}^{\text{spk}}$ for the EPSC trains (317Hz, green asterisk).

To test whether subthreshold frequency preferences were still evident under more physiological conditions, we applied trains of simulated excitatory postsynaptic currents (EPSCs) in the full nonlinear model (example waveform in Fig 7A). As before, Max-min analysis was applied to generate impedance profiles for the full subthreshold range of EPSC amplitudes. The resulting EPSC-$f_{\text{res}}^{\text{sub}}$ were then compared to those observed for sinusoidal stimuli of equivalent relative amplitude (example waveform in Fig 7B). While subthreshold resonance was observed for EPSCs trains, impedance profiles appeared less band-pass compared to sinusoidal stimuli, especially at low stimulus amplitudes (Fig 7Ci and 7Cii). Despite this, the range of $f_{\text{res}}^{\text{sub}}$ associated with synaptic inputs (202-316Hz) was larger than for sinusoidal inputs (242-281Hz) (Fig 7C main panel). For suprathreshold EPSC trains (>1.84nA) an evoked resonant peak was also prominent and the EPSC- $f_{\text{res}}^{\text{spk}}$ (317Hz) closely matched the maximum EPSC- $f_{\text{res}}^{\text{sub}}$(316Hz). This meant that the $f_{\text{res}}^{\text{spk}}$ increased from 285Hz for sinusoidal stimuli (green asterisk Fig 6C) to 317Hz for EPSC train stimuli (green asterisk Fig 7D). In addition to having a V-shaped indicator for suprathreshold resonance, the heat map for EPSC trains also featured entrainment at low stimulus frequencies with an asymptotic threshold amplitude at zero frequency of approximately 3.1nA: the threshold value for a single EPSC to elicit a spike.

## Discussion

A major finding of this study is that a low-threshold, voltage-gated potassium current, I_KLT_, underlies a high-frequency (>100Hz) subthreshold resonance in the gerbil MSO. In particular, the voltage-gated conductance of I_KLT_ was key to the emergence of this resonance as freezing the I_KLT_ conductance at rest either experimentally or in the computational model abolished band-pass filtering altogether. These findings are consistent with a recent prediction that I_KLT_ is the ‘resonant’ current in the guinea pig MSO and highlight the importance of I_KLT_ to subthreshold signal processing of MSO neurons in low-frequency hearing animals [4]. In addition to characterizing this subthreshold resonance with small amplitude sinusoidal inputs, we found that the resonant frequency, sine-$f_{\text{res}}^{\text{sub}}$, increased with sinusoidal stimulus amplitude. This finding can not only be attributed to the amount of I_KLT_ activated but also to its accelerated activation (smaller time constant) at depolarized levels. Of unique significance, we found that as stimulus amplitude was increased adequately to evoke spiking that the sine-$f_{\text{res}}^{\text{sub}}$ approached the spike resonant frequency sine-$f_{\text{res}}^{\text{spk}}$. Previous studies have shown the importance of I_KLT_ to MSO neurons’ suprathreshold responses when sinusoidal stimuli are applied [20,22]; the linkage of their subthreshold and suprathreshold resonant properties stresses the critical role that I_KLT_ plays in MSO physiology in both regimes. Few studies of resonance have demonstrated the connection between sub- and supra-threshold features as we have done here. In addition to the larger sinusoidal amplitudes, trains of subthreshold and suprathreshold EPSCs were also presented to the full neuron model. While weak subthreshold EPSC trains were effectively low-pass filtered, resonant peaks with Q factors >1.1 were observed for subthreshold EPSCs >1.4nA. The maximum EPSC-$f_{\text{res}}^{\text{sub}}$ also closely matched the EPSC-$f_{\text{res}}^{\text{spk}}$, leading to a spike threshold for EPSC trains that was considerably less (41.5%) than for a single EPSC at zero frequency. This not only demonstrates the importance of stimulus waveform when measuring impedance in neurons but also highlights how resonant properties may also be effective in selecting frequencies of synaptic input.

### The subthreshold resonant properties of the MSO neuronal population

I_KLT_ is a major contributor to gerbil MSO principal neurons’ intrinsic properties at rest [11,14,27,29]. Given the significance of I_KLT_ for high-frequency subthreshold resonance described in this study, the correlation between sine-$f_{\text{res}}^{\text{sub}}$ and input resistance likely arises from different densities of I_KLT_ at rest across the MSO neuron population (Fig 2B). Although Q factor was also correlated to the input resistance, the relationship between sine-$f_{\text{res}}^{\text{sub}}$ and Q factor appeared less clear (Fig 2C). Analysis of subthreshold resonance in a linear model suggests that the ratio of “resonant” and “leak” currents may explain why the Q factor did not appear to increase concurrently with sine-$f_{\text{res}}^{\text{sub}}$ as the input resistance decreased [33]. While increasing either “resonant” or “leak” currents separately in the linear model raises both the sine-$f_{\text{res}}^{\text{sub}}$ and Q factor, increasing both currents together so that their ratio remains the same can produce an increase in the sine-$f_{\text{res}}^{\text{sub}}$ with little change in the Q factor (Fig 3B inset and 4B in [33]). In the MSO neuron model, the other major conductance at rest is the hyperpolarization-activated mixed cation current, I_H_ [17,29,34]. It displays a much slower activation time course than I_KLT_ and therefore effectively acts as the “leak" current for the purposes of sinusoidal stimuli faster than 10Hz [22,29]. Although it has been shown that I_H_ varies in density across the gerbil MSO nucleus [34], no evidence has yet been found for a corresponding change in I_KLT_ density [11,29].

Aside from heterogeneities in conductance densities, the range of temperatures at which electrophysiological recordings were performed here may have also influenced MSO neurons’ resonant properties by altering channel kinetics. When increasing the temperature from 32°C to 37°C (through 35°) in the full neuron model (as was the case experimentally, see Methods), both Q factor and sine-$f_{\text{res}}^{\text{sub}}$ were found to increase (at 32°C: Q factor = 1.79, sine-$f_{\text{res}}^{\text{sub}}$ = 211Hz; at 35°C: Q factor = 1.88, sine-$f_{\text{res}}^{\text{sub}}$ = 242Hz; at 37°C, Q factor = 2.02, sine-$f_{\text{res}}^{\text{sub}}$ = 272Hz). Given that the experimentally-derived Q factors spanned a larger range (from 1.045 to 1.905) and that a significant correlation between Q factors and sine-$f_{\text{res}}^{\text{sub}}$ was missing (Fig 2D), temperature may have influenced the dynamics but it alone cannot explain the variance in resonant properties we observed.

### Comparing subthreshold resonance to evoked spike resonance

Comparisons of subthreshold resonance with spike frequency preference have been made but mostly in a qualitative sense, relying on intuition based on small signal analysis rather than systematic tuning of input amplitude or an intrinsic property to test for linkage (see [34] for exception). Studies of low frequency subthreshold resonance have typically found similar frequency selectivity as for voltage-gated slow oscillations [2,25,35–36]. Similarity between subthreshold resonance and frequency preference of spiking was demonstrated for the Hodgkin-Huxley model by using large amplitude ZAP currents or by varying the time between paired brief inputs [9]. In early work, resonant-like behavior of the Hodgkin-Huxley model was compared with squid axon responses [37]. The analysis involved subthreshold damped oscillations in response to current steps; impedance curves were not obtained. It was also illustrated with the model that subthreshold resonant frequency increases with stimulus amplitude. However the standard Hodgkin-Huxley model is without I_KLT_ and it differs in excitability properties from MSO neurons and models. It displays tonic firing properties and its suprathreshold heat map will not be prominently V-shaped, as would be the case for neurons that fire tonically. The response map for periodic forcing can instead be very broad, including regions where the neuron or model responds with one or more spikes per cycle at low stimulus frequencies [20]. Unlike the standard Hodgkin-Huxley model, MSO principal neurons fire phasically; they spike more to an input’s rising slope i.e. the rate of depolarization, than to input amplitude or depolarization *per se*. Their phasic firing patterns lead to strong V-shaped heat maps observed in Fig 6D and 6E; blocking I_KLT_ with a pharmacological antagonist can lead to broader heat maps associated with tonic firing neurons like the Hodgkin-Huxley neuron model [20,22]. It therefore appears that I_KLT_ not only underlies the resonance we observed in the subthreshold regime but also the suprathreshold gating that generates a spike resonance for sinusoidal inputs.

**Fig 8:**
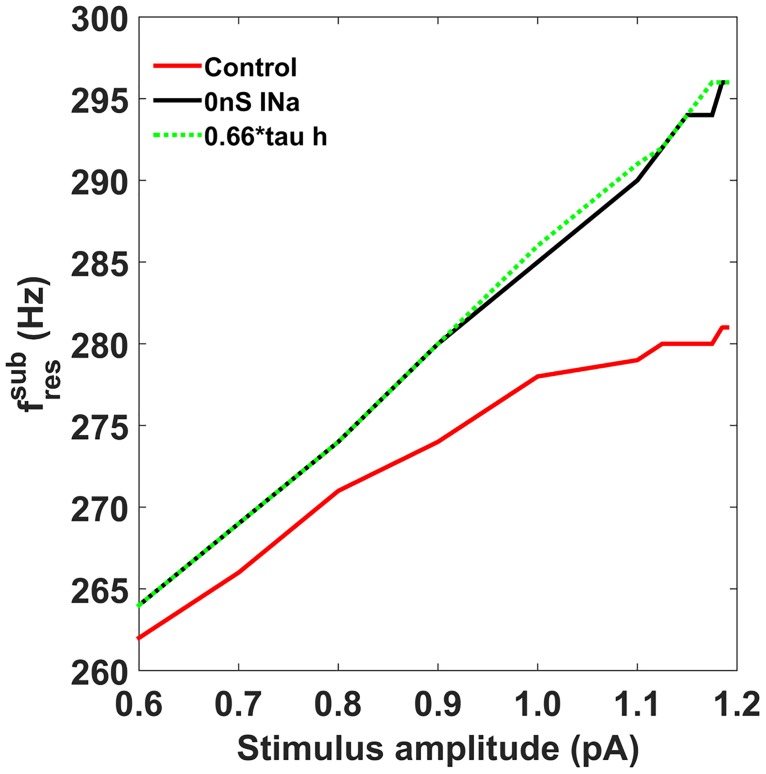
Inactivation of I_Na_ in the neuron model causes a plateau in sine-$f_{\text{res}}^{\text{sub}}$ at perithreshold amplitudes. Full nonlinear model shows plateauing in its sine-$f_{\text{res}}^{\text{sub}}$ at stimulus amplitudes above 0.8nA (red). Removing I_Na_ (black) or decreasing the inactivation time constant of I_Na_ by 34% (dotted green) was sufficient to recover a linear relationship between sine-$f_{\text{res}}^{\text{sub}}$ and stimulus amplitude.

Is I_KLT_ the only mechanism involved in MSO neuron excitability or is there another mechanism with a similar neural time scale? Sine-$f_{\text{res}}^{\text{sub}}$ appeared to diverge from a linear relationship at stimulus amplitudes above 1000pA in the full nonlinear model (Fig 8). At such high subthreshold amplitudes, significant I_Na_ inactivation is known to occur in MSO neurons [17–18]. Although I_Na_ is regenerative, it inactivates at comparatively low voltage in MSO neurons, contributes to phasic firing, and can play a role in subthreshold resonance [11,17,20,22]. Either accelerating inactivation of I_Na_ by 34% (dotted green, Fig 8) or removing I_Na_ altogether (black, Fig 8) in the neuron model proved sufficient to remove the plateau in sine-$f_{\text{res}}^{\text{sub}}$ observed in the full neuron model (red, Fig 8). It would therefore appear that two subthreshold resonant mechanisms occur at perithreshold amplitudes: inactivation of I_Na_ and I_KLT_ activation.

### Synaptic input and subthreshold resonance

I_KLT_ is also known to actively sculpt excitatory postsynaptic potentials (EPSPs) as they propagate from the dendrites to the somata of MSO neurons, resulting in somatic EPSPs with uniformly narrow half-widths [14,38]. Given this interaction, it was also of particular interest to understand how trains of brief synaptic input, a more physiologically relevant stimulus, could affect subthreshold and suprathreshold resonance in the MSO neuron model.

In the low-amplitude subthreshold regime, the differing impedance profiles for the sine-wave and EPSC trains (Fig 7C inset i and ii) were likely a result of how the fast, stereotyped synaptic waveform activated I_KLT_ differentially to the sinusoidal stimulus. Specifically, the fast rising slope of an ESPC does not vary with input frequency (whereas the slope increases with frequency for sinusoidal stimuli); therefore, trains of EPSCs could activate I_KLT_ more uniformly below the neuronal filter’s corner frequency (468Hz [$f_{corner}\;=\;\frac{1}{2\pi \tau_{m}}$]), leading to less band-pass filtering compared to the sinusoidal stimulus (Fig 7C inset i and dashed red line,Fig 7D). Additionally the lack of a hyperpolarizing component of the stimulus likely also contributed to the low-pass filtering of the simulated EPSCs at these low stimulus amplitudes. The push-pull of a sinusoidal drive appeared better matched to resonate with the steady state’s intrinsic damped oscillatory attraction in the subthreshold, linear regime than the one-sided, push-only (i.e. depolarizing) EPSC stimulus. Introducing inhibitory postsynaptic currents (IPSCs) which are appropriately timed relative to the EPSCs [26,39] could potentially provide the pull in the push-pull and consequently raise Q factors at these weak EPSC amplitudes closer to values observed for sinusoidal stimuli.

At larger subthreshold amplitudes, the missing hyperpolarizing components could explain not only the raised spike threshold for the simulated synaptic input (through the lack of I_KLT_ deactivation and deinactivation of I_Na_ [17]) but also the increased EPSC-$f_{\text{res}}^{\text{sub}}$ for perithreshold amplitudes (on average EPSCs are more depolarizing hence lead to greater and faster I_KLT_ activation). Interestingly, in the perithreshold EPSC regime, there was little evidence for EPSC-$f_{\text{res}}^{\text{sub}}$ plateauing as was the case for sinusoidal inputs (Fig 7C). This was likely due to the fast time course of ESPCs (as well as the absence of hyperpolarization) being temporally mismatched to I_Na_ inactivation. As a result, when I_Na_ was activated by EPSCs above 1.4nA, it only amplified the resonant peak causing Q factors to exceed 1.1 (Fig 7C inset ii and solid red line,Fig 7D).

In summary, comparing and interpreting impedance profiles generated by subthreshold sinusoidal inputs and trains of EPSCs highlights the importance of stimulus waveform. Even though the EPSC stimulus revealed low-pass rather than resonant properties in the weak amplitude limit, one should take care not to dismiss the possibility of resonance but consider the effects of larger amplitude. After all, the brief EPSC contains a mixture of many sinusoidal components with a broad amplitude distribution so it is not surprising that classical resonance might be masked. For increased amplitude, where the transition to frequency-dependent threshold for spike generation can be characterized, the EPSC captured slope-sensitivity of MSO neurons better than the sinusoidal waveform. That is, a sinusoidal input will reflect slope sensitivity only if the input amplitude is increasingly large for lower frequency.

### The functional impact of high-frequency resonance in the MSO

Based on the low-pass behavior for trains of weak EPSCs, we might have predicted erroneously that trains of brief EPSCs would not lead to spike resonance. Therefore a very interesting impact of high-frequency resonance on synaptic integration in the gerbil MSO model was the emergence of a prominent EPSC spike resonance (Fig 7D). Despite the fast-rising slope of an EPSC being invariant to input rate, the input threshold for spiking decreased from 3.14nA for a single EPSC to 1.84nA for an ESPC train at the EPSC-$f_{\text{res}}^{\text{spk}}$ (317Hz) (Fig 7D). Functionally this suggests that the prior history of synaptic input and its resulting I_KLT_/I_Na_ activation can actually alter an MSO neuron’s instantaneous slope-sensitivity and this may affect binaural tuning properties in MSO [38]. Possessing a dynamic threshold may appear counter-intuitive in neurons famed for their ability to process high-frequency acoustic inputs on a cycle-by-cycle basis [5–7]; however it potentially offers a means of counteracting synaptic depression to maintain spike probability across an ongoing input train: a phenomenon that has been previously observed for trains of subthreshold EPSCs [29].

While ESPC spike resonance was evident in our current study, it could not have influenced the computational finding of Remme et al. [4] that high-frequency resonance facilitates spatial cue extraction. Their model MSO neuron used a linear approximation of a “resonant” current and applied a fixed slope threshold for spiking (I_Na_ was absent so the model lacked an “amplifying” current) and therefore could not have displayed EPSC spike resonance [4]. Indeed if impedance profiles were generated using trains of EPSCs with the linearized model, we expect that low-pass filtering would be observed essentially across all subthreshold EPSC amplitudes. This feature supports the argument that a high-frequency (400Hz) subthreshold resonance may not be necessary to perform ITD processing in MSO neurons but the resonance may merely accompany the fast membrane properties underlying their phasic, slope-detecting behavior. Nevertheless we do not conclude that resonance has no role to play in the gerbil/guinea pig MSO *in vivo*. We believe that EPSC spike resonance merits further research: especially when considering the presentation of physiologically realistic synaptic trains to MSO neurons. Such investigation may involve introducing IPSCs to the synaptic train [26,38] as well as randomizing stimulus amplitude and jittering summed input [31,38]. In summary, our findings of spike resonance for brief EPSC trains (and in tandem an input threshold considerably lower than the single EPSC threshold) leave open some interesting questions about intrinsically-based preference for successively-timed EPSCs in MSO as well as other phasic firing systems.

### Closing thought

From a generalist’s viewpoint of excitability, the descriptor, “resonator”, is often associated with a system that fires repetitively over a narrow frequency range for steady input and entrains 1:1 to periodic stimulation around the resonant frequency. In contrast, an “integrator” can fire over a large frequency range with frequency that increases smoothly with stimulus amplitude and can fire by summating brief inputs of variable timings [40]. Phasic systems do not fire for steady or slowly varying inputs; yet in our study of a strongly phasic system (not a resonator as described above), we find spike resonance and resonance for weak sinusoids (classical small signal resonance). There’s motivation here for future work.
